# An overview on the successes, challenges and future perspective of a national school-based surveillance program: the CASPIAN study

**DOI:** 10.1186/s40200-014-0120-3

**Published:** 2014-12-20

**Authors:** Zeinab Ahadi, Gita Shafiee, Mostafa Qorbani, Sima Sajedinejad, Roya Kelishadi, Seyed Masoud Arzaghi, Bagher Larijani, Ramin Heshmat

**Affiliations:** Chronic Diseases Research Center, Endocrinology and Metabolism Population Sciences Institute, Tehran University of Medical Sciences, Tehran, Iran; Department of Public Health, Alborz University of Medical Sciences, Karaj, Iran; National Professional Officer, World Health Organization Office in Iran, Tehran, Iran; Pediatrics Department, Child Growth and Development Research Center and Faculty of Medicine, Isfahan University of Medical Sciences, Isfahan, Iran; Elderly Health Research Center, Endocrinology and Metabolism Population Sciences Institute, Tehran University of Medical Sciences, Tehran, Iran; Endocrinology and Metabolism Research Center, Endocrinology and Metabolism Clinical Sciences Institute, Tehran University of Medical Sciences, Tehran, Iran

**Keywords:** Prevention, School- based survey, Non-communicable diseases, Children, Adolescents

## Abstract

The Childhood and Adolescence Surveillance and PreventIon of Adult Non-communicable disease (CASPIAN) study is implemented in the Islamic Republic of Iran from 2003. The aim of this national school- based surveillance program was to provide accurate data of regular surveys of this program to be reviewing methodology, protocols, data collection and questionnaires of these surveys. Information was obtained from articles and books were published from CASPIAN studies. The CASPIAN studies were repeated every two years, with blood sampling for biochemical factors every four years. Methods and questionnaires of all surveys were similar at their core level and some optional factors added in different surveys. The results of CASPIAN studies represent the public health of Iranian children and adolescents that are useful for policy makers and based on them, intervention programs can set in national and sub-national level.

## Introduction

The Global school-based student health survey (GSHS) is a school-based survey performed among adolescents, aged 13–17 years, to obtain accurate information on behavioral risk factors and protective factors. GSHS program can help countries to make and develop policies and school health programs as well as allow countries and international agencies to make comparisons between countries on prevalence of health behaviors and protective factors. Also, gathering data from this program can use to determine trends in prevalence of behavioral risk factors and protective factors by country for evaluation of school health programs [1].

The self-administered questionnaire is used by GSHS program to gather information on behavioral risk factors and protective factors are in relationship with morbidity and mortality among children and youth [2]. The self-administered questionnaire included core questionnaire modules, core-expanded questions and country-specific questions that countries make their unique questionnaire for adolescents using the three components.

The 10 core questionnaire modules included alcohol use, tobacco use, drug use, sexual behaviors, hygiene, dietary behaviors, mental health, physical activity, protective factors, Violence and unintentional injury [1].

In 2001, WHO, in collaboration with UNAIDS, UNESCO, and UNICEF, and with technical assistance from the US Centers for Disease Control and Prevention (CDC), initiated development of the GSHS.

Since 2003, Ministries of Health and Education around the world have been using the GSHS to periodically monitor the prevalence of important health risk behaviors and protective factors among students. To date, several countries have completed a GSHS [3].

Therefore, for the first time in the Islamic Republic of Iran, a project has been initiated to provide a culturally-appropriate model for action-oriented interventions, and to implement a national school-based surveillance system for risk behaviors and risk factors for chronic diseases. The **C**hildhood and **A**dolescence **S**urveillance and **P**revent**I**on of **A**dult **N**on-communicable Disease (CASPIAN) Study is a joint collaboration of the WHO Regional Office for the Eastern Mediterranean (WHO/EMRO) and the Iranian Ministry of Health and Medical Education and the Ministry of Education [4]. To date, 4 phases of CASPIAN have completed. The surveys were repeated every 2 years, with blood sampling for biochemical factors every 4 years.

The aim of the current paper on this national school- based surveillance program is to provide accurate data of 4 surveys of this program to be reviewing methodology, protocols, data collection and questionnaires of these surveys and preparing a list of lessons learned from these studies for further researches.

## Methods and materials

We reviewed the documents and four surveys of this program in terms of methodology, protocols, data collection and questionnaires.

Reference lists of included articles and books were published from CASPIAN studies. The articles were identified through a search of Google scholar and PubMed databases that were published between 2006 and 2013.

Finally, we prepared recommendations and a list of lessons learned from these studies for further researches to design and implement necessary interventions in the next phases of the study.

## Conclusion

### CASPIAN I

This survey was conducted from 2003–2004 in rural and urban areas of central counties of 23 provinces in the Islamic Republic of Iran [4].

### CASPIAN II

This study was in 2007–2008 in rural and urban areas of 28 provinces [5].

### CASPIAN III

This study was performed from 2009–2010 in rural and urban areas of the central counties of 27 provinces [6].

### CASPIAN IV

This survey was conducted in 2011 and 2012 in rural and urban areas of the central counties of 31 provinces [7].

## Study population and sample size

### CASPIAN I

The sample size was calculated as 900 students, aged 6–18 years, in each district of the 23 provinces, taking 150 students of each sex and in each of the 3 age groups under study. Therefore, the total sample size was calculated to be 20, 700 in the 23 provinces, which was increased to 22, 000 to allow for a 5% estimated missing data. The students were selected from elementary, middle and high school by multistage, random cluster sampling from different residence (urban/rural) and different types of schools (public/private) to avoid socioeconomic bias [4,8].

### CASPIAN II

The final sample size was calculated as 9,216 students aged 11–18 years in the 28 districts under study, which was increased to 10,200 to allow with an estimated response rate of 85 percent (85 clusters of 120 people, 20 students in each sex and age group). The students were selected by multistage, random cluster sampling from last year of elementary school, middle and high school as well as different residence (urban/rural) [5].

### CASPIAN III

The total sample size in the 27 provinces was calculated to be 4,950, which was increased to 6,000 to allow with an estimated response rate of 80 percent. The survey was performed on 5,570 students, aged 10–18 years, who were selected by multistage random cluster sampling (84 clusters of 72 people, 12 students in each sex and age group). The schools were stratified according to location (urban/rural), and different types of schools (public/private) to avoid socioeconomic bias [6].

### CASPIAN IV

The final sample size was calculated as 14,880 students aged 6–18 years in the 30 provinces in Iran (48 cluster of 10 people in each province). The sampling method was a multistage cluster sampling. The students were selected from elementary, intermediate and high school by multistage, random cluster sampling from different residence (urban/rural) and different types of schools (public/private) [7].

## Instrument and measurement

All methods and questionnaires of CASPIAN studies were similar at their core and some factors add as an optional in different CASPIAN studies. In all CASPIAN surveys, a written informed consent and verbal consent was obtained from the parents and students; respectively, after complete description of the procedure involved.

### CASPIAN I

#### Questionnaire

The questionnaires were prepared based on the WHO Global School Health Survey (GSHS) and the WHO STEPwise approach to non-communicable diseases (Tools, version 9.5) that include core questionnaire modules, core-expanded questions and country specific questions that are composed to form a self-administered questionnaire.

A panel of experts affirmed the validity of the content. Item analysis and reliability measures were assessed based on a pilot study [4,9].

The questionnaire included the socio-demographic variables, family history of NCD (premature CVD, osteoporosis, obesity and cancer), as well as family dietary habits were contained in the parents’ questionnaire. In addition, students filled a food frequency questionnaire. The students’ self-reported physical activity pattern was assessed using a scaled questionnaire. All the questionnaires were modified and validated in Iranian youth [10,11].

Dietary and exercise habits were assessed for 21,111 school students (96% participation rate) ,aged 6–18 years, living in urban and rural areas.

In the 20 provinces which agreed to include a smoking questionnaire, the smoking habits (active/passive) were assessed with an anonymous questionnaire among 11,966 middle- and high school students [4].

#### Physical and laboratory measurements

All instruments were standardized before the examination, and the balance were zero-calibrated. In schools, trained nurses recorded dates of birth, and measured height and weight twice. Waist circumference (WC) and hip circumference (HiC) was measured as well as the blood pressure (BP) according to a standard protocol. Physical measures made for 21,111 school students (96% participation rate), aged 6–18 years, living in urban and rural areas.

For blood sampling, students were invited to attend the nearest health center to the school, accompanied by a parent. Fasting blood sugar (FBS), high-density lipoprotein cholesterol (HDL-C) and triglycerides (TG) and low-density lipoprotein-cholesterol (LDL-C) were measured. Lab measurements were performed in a subsample of 4,811 students aged 6–18 years in 6 provinces with a population of different ethnicities [4]. 1,000 subsamples of blood were stored for later lab measurements.

### CASPIAN II

#### Questionnaire

The questionnaires were prepared in Farsi based on Global School Health Survey (GSHS) and Youth Risk Behavior Surveillance (YRBS). The face validity was affirmed based on a pilot study. GSHS questionnaire was used for 3 age group but YRBS questionnaire used for middle- and high-school students.

The questionnaire included questions about socio-demographic characters, family history of NCD, as well as questions about the relationship with peers, psychosocial status of school, physical activity pattern, hygiene, violence and unintentional injury, protective factors, mental health, tobacco use and sexual behaviors.

Under the supervision of expert health care professionals, the students filled out the self-administered questionnaire. The questionnaires were filled with 9,171 school students [5].

#### Physical measurements

In this study height and weight only was measured [5].

### CASPIAN III

#### Questionnaire

The questionnaires were used in Farsi based on and the WHO global school-based student health survey, and added some more questions to the questionnaires of parents. The validity of their content was affirmed based on observations of an experts’ panel and item analysis. Reliability measures were assessed based on a pilot study (methodology).

The questionnaire of this study was similar to the CASPIAN II study. Similar other CASPIAN studies, under the supervision of expert health care professionals, the students filled out the self-administered questionnaire and the parent of student filled the questionnaire at home. 5,570 school students were assessed ( 95.6% participation rate) [6].

#### Physical and lab measurements

In the third survey of CASPIAN study, similar to CASPIAN I, anthropometric index, blood pressure and blood sampling (FBS and lipid profile) was measured, in addition liver enzymes such as Serum Glutamic OxaloaceticTransaminase(SGOT) and Serum Glutamic Pyruvate Transaminase (SGPT) was measured for liver function test.

Blood pressure and blood sampling were measured in a subsample of 5,408 and 4,790 students, respectively [6]. 4,000 subsamples of blood were stored for later lab measurements.

### CASPIAN IV

#### Questionnaire

The Questionnaire of students was similar the CASPIAN II and III. The questionnaire of parents had 7 sections included the results of inquiry, demographic variables, history of the student, family history, diet and unintentional injury of the student (90.6% participation rate) [7].

#### Physical measurements

In this study similar to CASPIAN III, height, weight, waist circumference, hip circumference and blood pressure were measured. In addition, the wrist circumference was measured [7].

#### Quality control

##### *CASPIAN I*

Data for all forms and questionnaires were entered twice and checked for completeness and inconsistencies. The supervisor of the data entry and analysis team quantified questionnaire data, including observations, interviewing, physical and laboratory measurements, and entered them into a computerized database (Microsoft Access XP). Data checking process was performed first at the district level by local supervisors to minimize missing and doubtful data, then at the national level by a group of statisticians. The quality of the data collected was monitored by the Data and Safety Monitoring Board (DSMB) of the project in order to identify and document problems in data quality [4]. The individual data and aggregated is available.

### CASPIAN II

For entering data, the questionnaires were gathered in the department of Health, Tehran University of medical sciences. Data were entered into a computerized database (Microsoft Excel XP).

Due to the large project and resource constraints, data were not entered twice. For quality control of data, we used two methods, the frequency of all data had taken and regarding to their limits all pert data had revised and had corrected. The 5 controlling question had chosen and if someone had been responding incorrectly to 3 questions from 5, his all data element [5]. The individual data is unavailable and aggregated is available.

### CASPIAN III

Data for all forms and questionnaires were gathered in the office of school health, ministry of health and medical education and entered into a computerized database.

Data checking was conducted first at the district level by local supervisors to minimize missing and doubtful data, then at the national level by a group of statisticians. They evaluated the databank fields for outliers and rechecked a sample of entering questionnaires for each operator [6]. The individual data and aggregated is available.

### CASPIAN IV

Data checking was conducted first at the district level by academic supervisors (Expert of school health). Then the data were controlled by national supervisors and operator [7].

The individual data and aggregated is available.

## Funding

Grant TSA03/11 World Health Organization/Eastern Mediterranean region and the Iranian Ministry of Health and the Ministry of Education funded the CASPIAN I study [12]. CASPIAN II, III and IV studies were funded by Iranian Ministry of Health.

Table 1 shows the summary of CASPIAN studies comparison.

**Table 1:** **Summary of CASPIAN studies’ comparison**

	**CASPIAN I**	**CASPIAN II**	**CASPIAN III**	**CASPIAN IV**
**Date of study**	**2003-2004**	**2007-2008**	**2009-2010**	**2011-2012**
Number of surveyed provinces	23	28	27	30
Sample size	21,111	9,171	5,570	13,486
Age range of students	6-18	11-18	10-18	6-18
Sampling methods	Multi-stage cluster sampling	Multi-stage cluster sampling	Multi-stage cluster sampling	Multi-stage cluster sampling
Types of schools	Public/Private	-	Public/Private	Public/Private
Location of schools	Urban/Rural	Urban/Rural	Urban/Rural	Urban/Rural
Generalization	Township	-	Township	Township
Used questionnaires	WHO STEPwise+ WHO Global School Health Survey	Youth Risk Behavior Surveillance (YRBS)+ WHO Global School Health Survey	WHO Global School Health Survey	Similar to CASPIAN III study
Dimension of self reported questionnaires	1. Socio-demographic/family history questionnaire/ birth weight and feeding in infancy	1.Student questionnaire included:	1. Student questionnaire	1. Student questionnaire
		a. Relationship with peers	Similar to CASPIAN II study except sexual behaviors	Similar to CASPIAN III study
		b. Psychosocial status of school		
		c. Dietary behaviors		
		d. Hygiene		
		e. Physical activity		
		f. Violence and unintentional injury		
		g. Protective factors		
		h. Mental health		
		l. Tobacco use		
		m. Sexual behaviors		
	2. Students dietary habits		2. Parents questionnaire included:	2. Parents questionnaire
				Similar to CASPIAN III study
	3. Physical activity questionnaire		a. Socio-demographic	
			b. Birth weight and feeding in infancy	
			c. family history	
			d. Dietary behaviors	
			e. Violence and unintentional injury about student	
	4. Students knowledge and attitude about CNCD-related risk behaviors questionnaire			
	5. Parental knowledge and attitude questionnaire			
Physical measurements	Height, weight, waist and hip circumference, blood pressure	Height and weight	Similar to CASPIAN I study except hip circumference	Similar to CASPIAN I study + wrist circumference
Lab measurements	FBS,TC, HDL-C,TG,LDL-C	-	Similar to CASPIAN I study+ liver function tests (SGOT,SGPT)	-
Quality control level	-At the district level by local supervisors	-	Similar to CASPIAN I study	- At the district level by academic supervisors
	-At the national level by a group of statisticians			- National supervisors and operator
	-Evaluation the databank fields for outliers			
Knowledge dissemination	One book [4] + 20 articles [8-26]	One book [5]	One book [6] + 28 articles [27-54]	Book is under preparation + 9 article [55-63]

### Future perspective

The CASPIAN studies have been provided data about public health of Iranian children and adolescent and represented health status in country; as a result it is so serious to repeat this study in the future. It is better that CASPIAN study will be done in a large number of participants in provinces of Iran, and the intervention will be designed based on the data of each province.

### Lessons learned

In the CASPIAN I study, the impact of after-school physical activity program on health-related fitness of mother/daughter pairs was investigated. The multicenter was performed from 2006–2007 in 7 provinces in Iran. Girl students (7^th^-10^th^ grade) and their mothers were selected by random cluster sampling. After school aerobic physical activity were held in 24 sessions, twice per week, during 90 minutes for 3 months. At the beginning and at the end of the study, physical activity, body weight, waist (WC) and hip circumference, body mass index (BMI), resting heart rate, cardio-respiratory fitness, flexibility and muscle strength were measured. After the intervention, mean of weight, BMI, WC and waist to hip ratio (WHR) decreased significantly among girls and their mothers [13]. Resting heart rate decreased significantly in both of them. Upper body and abdominal muscular strength and flexibility increased significantly at the end of study among students and their mothers [14]. The success of this trial might e a result of contribution of the student’s mothers in the physical activity program.

### Knowledge dissemination

One book and 20 articles were published from CASPIAN I study. One book was published from CASPIAN II study and from CASPIAN III, one book and 25 articles was available. The book of CASPIAN IV is under preparation and 8 articles were published.

## Recommendation

Quality control is codified for a study that included quality validation, trending, sampling, data management, data reporting, analysis and doing interventions.

These surveys reinforce the need to establish and reinforce intervention programs which are not only school- based but would involve the family structures, community prevention programs and government agencies to help preventing risky behaviors.

Instruments and methodology of these studies become more consistent with WHO Global School Health Survey. As a result, the results of these studies become comparable with the results of other countries.

## Summary

According to necessity of having information related to behaviors process and health risk factors related to students’ health, the CASPIAN study conducted at 2003–2004 and performed every 2 years. Until now, 4 phases of CASPIAN study was performed at I.R Iran. All methods and questionnaires of CASPIAN studies are similar at their core level and some factors add as an optional in different CASPIAN studies. The results of CASPIAN studies are useful for policy makers and based on them, intervention programs for improving students’ health will be planed.

## References

[1] **Global school-based student health survey (GSHS) purpose and methodology.** [http://www.who.int/chp/gshs/methodology/en/index.html]

[2] **Global school-based student health survey (GSHS).** [http://www.who.int/chp/gshs/en/index.html]

[3] Lasserre AM, Viswanathan B, Bovet P (2008). Global School-based Student Health Survey.

[4] Kelishadi R, Motlagh ME, Ardalan G, Zia-edini H, Riazi M, Arabi M, Barakati H: *Prevention of Chronic Non-communicable Diseases: Today Better than Tomorrow, CASPAIN Study.* Isfahan University of Medical Sciences Press; 2008.

[5] 5. Amirkhani A, Motlagh ME, Sedaghat M, Namazi R, Ardalan G, Haghdust A, Zia-edini H, Taslimi M, kamali K: *Health Status of Iranian students According to the Health-Risk Behaviors Survey 2006–2007.* Isfahan University of Medical Sciences Press; 2009.

[6] Motlagh ME, Kelishadi R, Heshmat R, Aminaei T, Dashti M, Ardalan G (2011). Health-Risk Behaviors Survey among Iranian Students 2009–2010.

[7] Kelishadi R, Heshmat R: **Childhood and Adolescence Surveillance and Prevention of Adult Non-communicable disease (Protocol).** In 2011–2012.

[8] Motlagh M, Kelishadi R, Ardalan G, Gheiratmand R, Majdzadeh R, Heidarzadeh A (2009). **Rationale, methods and first results of the Iranian national programme for prevention of chronic diseases from childhood: CASPIAN Study**. East Mediterr Health J.

[9] Kelishadi R, Ardalan G, Gheiratmand R, Majdzadeh R, Hosseini M, Gouya M, Razaghi E, Delavari A, Motaghian M, Barekati H (2008). **Thinness, overweight and obesity in a national sample of Iranian children and adolescents: CASPIAN Study**. Child Care Health Dev.

[10] Kelishadi R, Gouya MM, Adeli K, Ardalan G, Gheiratmand R, Majdzadeh R, Mahmoud-Arabi MS, Delavari A, Riazi MM, Barekati H (2008). **Factors associated with the metabolic syndrome in a national sample of youths: CASPIAN Study**. Nutr Metab Cardiovasc Dis.

[11] Kelishadi R, Ardalan G, Gheiratmand R, Gouya MM, Razaghi EM, Delavari A, Majdzadeh R, Heshmat R, Motaghian M, Barekati H (2007). **Association of physical activity and dietary behaviours in relation to the body mass index in a national sample of Iranian children and adolescents: CASPIAN Study**. Bull World Health Organ.

[12] Kelishadi R, Amirkhani A, Ardalan G, Ziaoddini H, Majdzadeh R (2009). An overview of a national surveillance program in iran for prevention of chronic non-communicable diseases from childhood: CASPIAN study. Iran J Public Health.

[13] Kelishadi R, Ziaee V, Ardalan G, Namazi A, Noormohammadpour P, Ghayour-Mobarhan M, Poursafa P (2010). A national experience on physical activity initiatives for adolescent girls and their mothers: CASPIAN study. Iran J Pediatr.

[14] Kargarfard M, Kelishadi R, Ziaee V, Ardalan G, Halabchi F, Mazaheri R, Poursafa P, Hayatbakhsh MR (2012). **The impact of an after-school physical activity program on health-related fitness of mother/daughter pairs: CASPIAN study**. Prev Med.

[15] Kelishadi R, Gheiratmand R, Ardalan G, Adeli K, Mehdi Gouya M, Mohammad Razaghi E, Majdzadeh R, Delavari A, Shariatinejad K, Motaghian M (2007). Association of anthropometric indices with cardiovascular disease risk factors among children and adolescents: CASPIAN study. Int J Cardiol.

[16] Kelishadi R, Ardalan G, Gheiratmand R, Adeli K, Delavari A, Majdzadeh R (2006). Paediatric metabolic syndrome and associated anthropometric indices: the CASPIAN study. Acta Paediatr.

[17] Kelishadi R, Gouya MM, Ardalan G, Hosseini M, Motaghian M, Delavari A, Majdzadeh R, Heidarzadeh A, Mahmoud-Arabi MS, Riazi MM (2007). **First reference curves of waist and hip circumferences in an Asian population of youths: CASPIAN study**. J Trop Pediatr.

[18] Kelishadi R, Ardalan G, Gheiratmand R, Majdzadeh R, Delavari A, Heshmat R, Reza Mokhtari M, Mohammad Razaghi E, Motaghian M, Ahangar-Nazari I (2006). **Smoking behavior and its influencing factors in a national-representative sample of Iranian adolescents: CASPIAN study**. Prev Med.

[19] Kelishadi R, Cook SR, Motlagh ME, Gouya MM, Ardalan G, Motaghian M, Majdzadeh R, Ramezani MA (2008). Metabolically obese normal weight and phenotypically obese metabolically normal youths: the CASPIAN study. J Am Diet Assoc.

[20] Kelishadi R, Ardalan G, Gheiratmand R, Majdzadeh R, Delavari A, Heshmat R, Gouya MM, Razaghi EM, Motaghian M, Mokhtari MR (2006). **Blood pressure and its influencing factors in a national representative sample of Iranian children and adolescents: the CASPIAN study**. Eur J Cardiovasc Prev Rehabil.

[21] Kelishadi R, Ardalan G, Adeli K, Motaghian M, Majdzadeh R, Mahmood-Arabi MS, Delavari A, Riazi MM, Namazi R, Ramezani MA (2007). **Factor analysis of cardiovascular risk clustering in pediatric metabolic syndrome: CASPIAN study**. Ann Nutr Metab.

[22] Alavian S-M, Motlagh ME, Ardalan G, Motaghian M, Davarpanah AH, Kelishadi R (2008). **Hypertriglyceridemic waist phenotype and associated lifestyle factors in a national population of youths: CASPIAN study**. J Trop Pediatr.

[23] Kelishadi R, Ardalan G, Gheiratmand R, Ramezani A (2006). **Is family history of premature cardiovascular diseases appropriate for detection of dyslipidemic children in population-based preventive medicine programs? CASPIAN study**. Pediatr Cardiol.

[24] Ziaee V, Kelishadi R, Ardalan G, Gheiratmand R, Majdzadeh S, Monazzam M (2006). **Physical activity in Iranian students CASPIAN study**. Iran J Pediatr.

[25] Kelishadi R, Ardalan G, Gheiratmand R, Sheikholeslam R, Majdzadeh S, Delavari A, Monazzam M, Barakati S, Heshmat R (2005). **Do the dietary habits of our community warrant health of children and adolescents now and in future? CASPIAN study**. Iran J Pediatr.

[26] Kelishadi R, Ardalan G, Gheyratmand R, Sheikh al-Islam R, Majdzadeh S, Delawari A (2005). Can the dietary habits of our society provide future health of children and adolescents? CASPIAN study. Iran J Pediatr.

[27] Khashayar P, Heshmat R, Qorbani M, Motlagh ME, Aminaee T, Ardalan G, Farrokhi-Khajeh-Pasha Y, Taslimi M, Larijani B, Kelishadi R: **Metabolic syndrome and cardiovascular risk factors in a national sample of adolescent population in the middle east and north Africa: the CASPIAN III study.** *Int J Endocrinol* 2013, **2013**:Article ID 702095, 702098 pages. 10.1155/2013/702095 PMC3580930 23476647

[28] Mansourian M, Marateb HR, Kelishadi R, Motlagh ME, Aminaee T, Taslimi M, Majdzadeh R, Heshmat R, Ardalan G, Poursafa P (2012). **First growth curves based on the World Health Organization reference in a Nationally-Representative Sample of Pediatric Population in the Middle East and North Africa (MENA): the CASPIAN-III study**. BMC Pediatr.

[29] Kelishadi R, Gheissari A, Bazookar N, Motlagh ME, Taslimi M, Ardalan G (2013). Kidney function in obese adolescents with or without metabolic syndrome in a nationally-representative sample of pediatric population: first report from the Middle East and North Africa: the CASPIAN-III study: a case–control study. J Res Med Sci.

[30] Izadi V, Kelishadi R, Qorbani M, EsmaeilMotlagh M, Taslimi M, Heshmat R, Ardalan G, Azadbakht L (2013). **Duration of breast-feeding and cardiovascular risk factors among Iranian children and adolescents: the CASPIAN III study**. Nutrition.

[31] Kelishadi R, Abtahi S-H, Qorbani M, Heshmat R, Motlagh ME, Taslimi M, Aminaee T, Ardalan G, Poursafa P, Moin P (2012). First National report on Aminotransaminases’ percentiles in children of the Middle East and North Africa (MENA): the CASPIAN-III study. Hepat Mon.

[32] Kelishadi R, Ardalan G, Motlagh ME, Shariatinejad K, Heshmat R, Poursafa P, Fakhri M, Tajadini M, Taslimi M (2014). National report on the association of serum vitamin D with cardiometabolic risk factors in the pediatric population of the Middle East and North Africa (MENA): the CASPIAN-III study. Nutrition.

[33] Kelishadi R, Mirmoghtadaee P, Qorbani M, Motlagh ME, Heshmat R, Taslimi M, Mahmoudarabi M, Ardalan G, Larijani B (2013). **Tooth brushing and cardiometabolic risk factors in adolescents: is there an association? The CASPIAN-III study**. Int J Prev Med.

[34] Mohammadi F, Qorbani M, Kelishadi R, Baygi F, Ardalan G, Taslimi M, Mahmoudarabi M, Motlagh M-E, Asayesh H, Larijani B (2014). Association of cardiometabolic risk factors and hepatic enzymes in a national sample of Iranian children and adolescents: the CASPIAN-III study. J Pediatr Gastroenterol Nutr.

[35] Shafiee G, Kelishadi R, Heshmat R, Qorbani M, Motlagh ME, Aminaee T, Ardalan G, Taslimi M, Poursafa P, Larijani B (2013). **First report on the validity of a continuous Metabolic Syndrome score as an indicator for Metabolic Syndrome in a national sample of paediatric population—the CASPIAN-III study**. Endokrynologia Polska.

[36] Jari M, Qorbani M, Motlagh ME, Heshmat R, Ardalan G, Kelishadi R (2014). **Association of overweight and obesity with mental distress in Iranian adolescents: the CASPIAN-III study**. Int J Prev Med.

[37] Kelishadi R, Askarieh A, Motlagh ME, Tajadini M, Heshmat R, Ardalan G, Fallahi S, Poursafa P (2013). Association of blood cadmium level with cardiometabolic risk factors and liver enzymes in a nationally representative sample of adolescents: the CASPIAN-III study. J Environ Public Health.

[38] Kelishadi R, Ataei E, Ardalan G, Nazemian M, Tajadini M, Heshmat R, Keikha M, Motlagh ME (2014). **Relationship of serum magnesium and vitamin D levels in a nationally-representative sample of Iranian adolescents: the CASPIAN-III study**. Int J Prev Med.

[39] Kelishadi R, Hajizadeh M, Ardalan G, Poursafa P, Fakhri M (2013). Association of Resistin and hs-CRP with Liver Enzymes and Components of the Metabolic Syndrome in Iranian Adolescents with Excess Weight: The CASPIAN-III Study.

[40] Kelishadi R, Motlagh ME, Roomizadeh P, Abtahi S-H, Qorbani M, Taslimi M, Heshmat R, Aminaee T, Ardalan G, Poursafa P (2013). **First report on path analysis for cardiometabolic components in a nationally representative sample of pediatric population in the Middle East and North Africa (MENA): the CASPIAN-III study**. Ann Nutr Metab.

[41] Kelishadi R, Marashinia F, Heshmat R, Motlagh M-E, Qorbani M, Taslimi M, Nourbakhsh M, Ardalan G, Poursafa P (2013). **First report on body image and weight control in a nationally representative sample of a pediatric population in the Middle East and North Africa: the CASPIAN-III study**. Arch Med Sci.

[42] Azadbakht L, Kelishadi R, Khodarahmi M, Qorbani M, Heshmat R, Motlagh ME, Taslimi M, Ardalan G (2013). **The association of sleep duration and cardiometabolic risk factors in a national sample of children and adolescents: the CASPIAN III study**. Nutrition.

[43] Azadbakht L, Kelishadi R, Saraf-Bank S, Qorbani M, Ardalan G, Heshmat R, Taslimi M, Motlagh ME (2014). **The association of birth weight with cardiovascular risk factors and mental problems among Iranian school-aged children: the CASPIAN-III study**. Nutrition.

[44] Gheissari A, Kelishadi R, Roomizadeh P, Abedini A, Haghjooy-Javanmard S, Abtahi S-H, Mehdikhani B, Shafiei M (2013). **Chronic kidney disease stages 3–5 in Iranian children: need for a school-based screening strategy: the CASPIAN-III study**. Int J Prev Med.

[45] Ataie-Jafari A, Heshmat R, Kelishadi R, Ardalan G, Mahmoudarabi M, Rezapoor A, Motlagh ME, Asayesh H, Larijani B, Qorbani M (2014). **Generalized or abdominal obesity: which one better identifies cardiometabolic risk factors among children and adolescents? The CASPIAN III study**. J Trop Pediatr.

[46] Qorbani M, Kelishadi R, Taheri E, Motlagh ME, Arzaghi SM, Ardalan G, Chinian M, Mahmoudarabi M, Rezapoor A, Asayesh H (2014). Association between psychosocial distress with cardio metabolic risk factors and liver enzymes in a nationally-representative sample of Iranian children and adolescents: the CASPIAN-III study. J Diabetes Metab Disord.

[47] Heshmat R, Kelishadi R, Motamed-Gorji N, Motlagh M-E, Ardalan G, Arifirad T, Rastad H, Asayesh H, Djalalinia S, Larijani B: **Association between body mass index and perceived weight status with self-rated health and life satisfaction in Iranian children and adolescents: the CASPIAN-III study.** *Qual Life Res* 2014, Jul 20. [Epub ahead of print]. 10.1007/s11136-014-0757-x 25038635

[48] Hosseini M, Maghami M, Kelishadi R, Motlagh ME, Khoshbin S, Amirkhani A, Heshmat R, Taslimi M, Ardalan G, Hosseini SM (2013). **First report on self-rated health in a Nationally-representative sample of Iranian adolescents: the CASPIAN-iii study**. Int J Prev Med.

[49] Poursafa P, Mansourian M, Motlagh M-E, Ardalan G, Kelishadi R (2014). **Is air quality index associated with cardiometabolic risk factors in adolescents? The CASPIAN-III study**. Environ Res.

[50] Rahmanian M, Kelishadi R, Qorbani M, Motlagh ME, Shafiee G, Aminaee T, Ardalan G, Taslimi M, Poursafa P, Asayesh H (2014). Dual burden of body weight among Iranian children and adolescents in 2003 and 2010: the CASPIAN-III study. Heart Dis.

[51] Bahrami E, Mirmoghtadaee P, Ardalan G, Zarkesh-Esfahani H, Tajaddini MH, Haghjooy-Javanmard S, Najafi H, Kelishadi R (2014). Insulin and leptin levels in overweight and normal-weight Iranian adolescents: the CASPIAN-III study. J Res Med Sci.

[52] Poursafa P, Ataee E, Motlagh ME, Ardalan G, Tajadini MH, Yazdi M, Kelishadi R (2014). Association of serum lead and mercury level with cardiometabolic risk factors and liver enzymes in a nationally representative sample of adolescents: the CASPIAN-III study. Environ Sci Pollut Res.

[53] Kelishadi R, Heshmat R, Motlagh ME, Majdzadeh R, Keramatian K, Qorbani M, Taslimi M, Aminaee T, Ardalan G, Poursafa P (2012). Methodology and early findings of the third survey of CASPIAN study: a national school-based surveillance of students’ high risk behaviors. Int J Prev Med.

[54] Ahadi Z, Qorbani M, Kelishadi R, Ardalan G, Taslimi M, Mahmoudarabi M, Motlagh ME, Asayesh H, Shafiee G, Larijani B, Heshmat R (2014). Regional disparities in psychiatric distress, violent behavior, and life satisfaction in Iranian adolescents: the CASPIAN-III study. J Dev Behav Pediatr.

[55] Kelishadi R, Ardalan G, Qorbani M, Ataie-Jafari A, Bahreynian M, Taslimi M, Motlagh ME, Heshmat R (2013). Methodology and early findings of the fourth survey of childhood and adolescence surveillance and prevention of adult non-communicable disease in Iran: the CASPIAN-IV study. Int J Prev Med.

[56] Kelishadi R, Majdzadeh R, Motlagh M-E, Heshmat R, Aminaee T, Ardalan G, Esmaillzadeh A, Azadbakht L, Poursafa P, Movahedian M (2012). Development and evaluation of a questionnaire for assessment of determinants of weight disorders among children and adolescents: the Caspian-IV study. Int J Prev Med.

[57] Kelishadi R, Qorbani M, Motlagh ME, Heshmat R, Ardalan G, Jari M (2014). Relationship between leisure time screen activity and aggressive and violent behaviour in Iranian children and adolescents: the CASPIAN-IV study. Paediatr Int Child Health.

[58] Kelishadi R, Qorbani M, Esmaeel Motlagh M, Ardalan G, Moafi M, Mahmood-Arabi M, Heshmat R, Jari M (2014). Frequency, causes, and places of unintentional injuries in a nationally representative sample of Iranian children and adolescents: the CASPIAN-IV study. Int J Prev Med.

[59] Sadinejad M, Kelishadi R, Qorbani M, Shahsanai A, Esmaeel Motlagh M, Ardalan G, Heshmat R, Keikha M (2014). A nationwide survey on some hygienic behaviors of Iranian children and adolescents: the CASPIAN-IV study. Int J Prev Med.

[60] Fallah Z, Qorbani M, Motlagh ME, Heshmat R, Ardalan G, Kelishadi R (2014). Prevalence of prehypertension and hypertension in a nationally representative sample of Iranian children and adolescents: the CASPIAN-IV study. Int J Prev Med.

[61] Jari M, Qorbani M, Motlagh ME, Heshmat R, Ardalan G, Kelishadi R (2014). A nationwide survey on the daily screen time of Iranian children and adolescents: the CASPIAN-IV study. Int J Prev Med.

[62] Zahedi H, Kelishadi R, Heshmat R, Motlagh ME, Ranjbar SH, Ardalan G, Payab M, Chinian M, Asayesh H, Larijani B (2014). Association of junk food consumption with mental health in a national sample of Iranian children and adolescents: the CASPIAN-IV study. Nutrition.

[63] Payab M, Kelishadi R, Qorbani M, Motlagh ME, Ranjbar SH, Ardalan G, Zahedi H, Chinian M, Asayesh H, Larijani B, Heshmat R (2014). Association of junk food consumption with high blood pressure and obesity in Iranian children and adolescents: the CASPIAN-IV study. J Pediatr (Rio J).

